# The complete chloroplast genome of *Ophioglossum vulgatum* L. (Ophioglossaceae) and phylogenetic analysis

**DOI:** 10.1080/23802359.2021.1966333

**Published:** 2021-08-24

**Authors:** Jing Hao, Yingyi Liang, Ming Zhu, Jingyao Ping, Peipei Feng, Yingjuan Su, Ting Wang

**Affiliations:** a College of Life Sciences, South China Agricultural University, Guangzhou, PR China; b School of Life Sciences, Sun Yat-sen University, Guangzhou, PR China; c Research Institute of Sun Yat-sen University in Shenzhen, Shenzhen, PR China

**Keywords:** Adder’s-tongue ferns, chloroplast genome, phylogenetic analysis

## Abstract

*Ophioglossum vulgatum* is a rare and ancient fern. In this study, the chloroplast (cp) genome of *O. vulgatum* was completely sequenced. The genome size is 138,562 bp, which contains a large single-copy (LSC) region with 99,351 bp, a small single-copy (SSC) region with 19,661 bp, and two inverted repeats (IR) regions of 9,775 bp each. Additionally, the overall GC content is 42.14%. It encodes a total 129 genes, including 84 protein-coding genes, 37 tRNA genes, and 8 rRNA genes. The Bayesian phylogenetic tree shows that *O. vulgatum* and *O. californicum* formed a monophyletic branch. This study can provide a molecular basis for studying the phylogenetic genomics and population variation of Ophioglossaceae.

*Ophioglossum vulgatum* (Ophioglossaceae) is a rare and ancient fern. It is mainly distributed in the northern hemisphere, generally growing in wet mountains, river banks, and ditches (Zhu et al. 2014). The whole plant can be used as medicine (Yang et al. 2019). It can clear away heat, diminish inflammation, and treat some cancers (Lu et al. 2009). It is known as the ‘Medicine King’ in Taiwan, China (Hu et al. 2016). In addition, it has important scientific research value in plant systematics and pteridophyte phylogeny (Zhu et al. 2014). The relationships among the branches of Ophioglossaceae were not well solved in previous molecular studies (Zhang, Fan, et al. 2020). Obtaining the entire chloroplast genome can provide a molecular basis for studying the phylogenetic genomics and population variation of Ophioglossaceae.

Fresh leaves were sampled from the campus of the South China Agricultural University (SCAU) (E113°20′, N23°9′). The specimen is stored in the Herbarium of South China Agricultural University (SCAUB, specimen code: JHao202105; herbaria acronyms follow Thiers 2021, continuously updated). Genomic DNA was extracted using Plant Genomic DNA Kit (Kangweishiji CW0553, China). Illumina Novaseq6000 (Illumina, San Diego, CA) high-throughput sequencing platform was used for sequencing, and the sequencing strategy was PE150 (Pair-End 150). Clean reads with high quality were obtained by filtering the original sequences. A total of 7,587,551 clean reads were generated. The chloroplast (cp) genome sequence was assembled using SPAdes version 3.5.0 (Bankevich et al. 2012). SPAdes with multi kmers from 79 to 97, and other paratemers were set default. We used CpGAVAS (Liu et al. 2012) and ORFfinder (National Library of Medicine, U.S., National Center for Biotechnology Information, 2004; cited 2021 April 25. Available from: https://www.ncbi.nlm.nih.gov/orffinder/) for cp genome annotation. For the preliminary annotation results, the methods of Blastn and Blastp (National Library of Medicine, U.S., National Center for Biotechnology Information, 2004; cited 2021 April 25. Available from: https://blast.ncbi.nlm.nih.gov/Blast.cgi/) were used to compare and verify the coding proteins and rRNAs of the cp genomes with related species. The annotation of tRNA was carried out by ARWEN (Laslett and Canback 2008). If abnormal tRNA occurred, tRNAscan-SE2.0 (Lowe and Chan 2016) was used to predict jointly, and finally, tRNAs with unreasonable length and incomplete structure were discarded. Fifteen species were selected to reconstruct the phylogenetic tree. The program MAFFT plugin (selected "-auto" strategy) in PhyloSuite version 1.2.1 (Zhang, Gao, et al. 2020) was used to create a multiple sequence alignment of the complete cp genome of *O. vulgatum* plus 14 other plants, in which the sequences were downloaded from GenBank (*Equisetum arvense* was selected as outgroup). GTR + F+I + G4 was chosen as the best-fit model according to the Bayesian Information Criterion (BIC). We reconstructed the Bayesian phylogenetic tree with PhyloSuite (2,000,000 generations, Nst = 6, rates = invgamma) (Figure 1).

**Figure 1.**
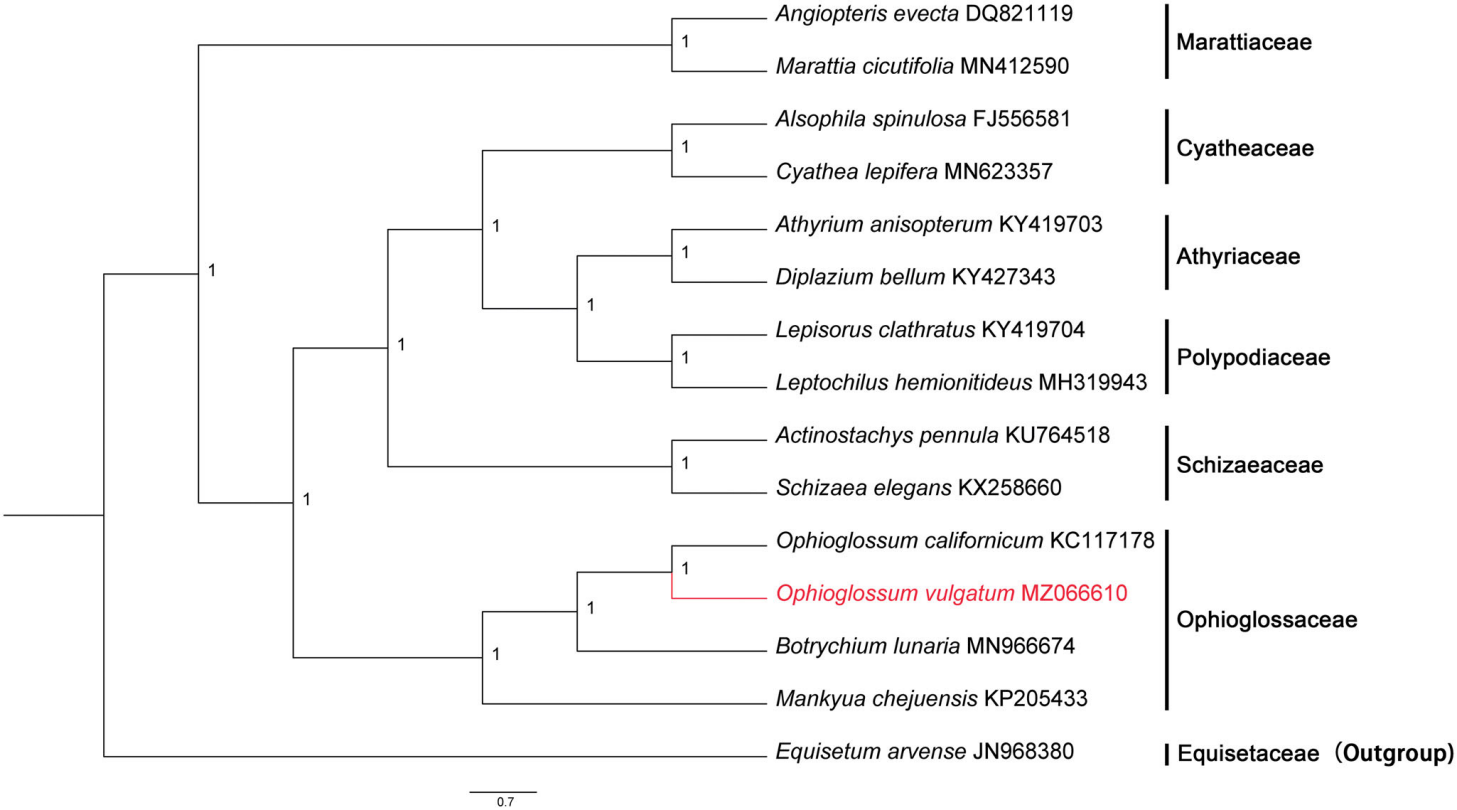
The Bayesian phylogenetic tree constructed based on the complete chloroplast genome sequences of 15 species. *Equisetum arvense* was selected as outgroup. The posterior probability of Bayesian was 1.

The complete cp genome of *O. vulgatum* is 138,562 bp in size with circular DNA molecular structure. It contains a large single-copy (LSC) region with 99,351 bp, a small single-copy (SSC) region with 19,661 bp, and two inverted repeats regions (IRs) of 9775 bp each. The overall GC content of this cp genome is 42.14%. The GC contents in LSC, SSC, and IR regions were 40.60%, 38.99%, and 53.14%, respectively. The cp genome encodes a total 129 genes, including 84 protein-coding genes, 37 tRNA genes, and 8 rRNA genes. Among these genes, eight genes (*atpF, ndhA, ndhB, rpl2, rpl16, petB, petD,* and *rpoC1*) contain one intron, while two genes (*clpP, ycf3*) have two introns. Additionally, the ratios of A, T, G, and C are 28.94%, 28.92%, 20.54%, and 21.59%. As shown in the Bayesian tree (Figure 1), the phylogenetic analysis shows that *O. vulgatum* and *O. californicum* formed a monophyletic branch. The cp genome of *O. vulgatum* can provide valuable genomic information to further phylogenetic relationship of Ophioglossaceae.

## Data Availability

The data that support the findings of this study are available in GenBank of NCBI at (https://www.ncbi.nlm.nih.gov/), reference number MZ066610. The associated BioProject, SRA, and Bio-Sample numbers are PRJNA739081, SRX11193688, and SAMN19771167, respectively.
